# Supplementary material for: Micronutrients and Breast Cancer Progression: A Systematic Review
Source: Nutrients. 2020 Nov 25;12(12):3613. doi: 10.3390/nu12123613 (PMC7759972; doi:10.3390/nu12123613)
Supplement: Supplementary file 1 [file nutrients-12-03613-s001.pdf]

SUPPLEMENTARY MATERIAL

Table S1. Vitamin D, Cell culture

AUTHOR & YEAR of PUBLICATION	MICRONUTRIENT STUDIED	MODEL	DOSIS & TIME	REFERENCE
**WILMANSKI 2017**	Vit D	MCF10A-ras & MCF10A-ErbB2	[1,25(OH)$_2$D] < 0.1 % in fresh medium changed every 24h	26
**PLUCHINO 2015**	Ergosterol	MCF10A & CF12A	10nM 24h	30
**WAHLER 2015**	Vit D	MCF10DCIS (basal-like BC)	1α25(OH)$_2$D$_3$: 100nM 24h	19
**ZHENG 2013**	Vit D (1,25(OH)$_2$D)	MCF10A & MCF10A-ras	1,25(OH)$_2$D 10nM for 4 days	29
**ZHOU 2016**	Vit D	Harvey-ras oncogene transformed MCF10A	1,25(OH)$_2$D 10nM in medium changed every 24h for 3 days	27
**SHAN 2017**	Vit D	Mammospheres of TNBC with CSC properties	10nM and/or 100nM 5 days	18
**GARCÍA-BECERRA 2010**	Vit D (Calcitriol)	Cell culture deviated from breast tumors needle biopsy & MDA-MB-231	24h incubation: 10^{-7}, 10^{-8}, 10^{-9} &10^{-10}M.	31
**PADUCH 2005**	Vit D	T47D in 2D & 3D models	10, 50 & 100nM/L 24 & 48 h	28
**TAVERA-MENDOZA 2017**	Vit D	MCF-7, ZR75, MDA-MB-231 & MCF10A	100nM, 4h	32
**KRISHNAN 2010**	Vit D (Calcitriol)	MCF-7, T47D, XR-75-1 & MDA-MB-231	24h: 10nM in all but MDA-MB-231: 100nM	17
**OOI 2010**	Vit D	MCF-7	1,25(OH)$_2$D$_3$: 10^{-7}, 10^{-8} o 10^{-9}M once a day for 6 days	24
**KOREN 2001**	Vit D (1,25(OH)$_2$D)	MCF-7	10-100 nM 24-48h	22
**YUAN 2012**	Vit D	MCF-7	100 nM/l 72h	20
**SWAMI 2000**	1,25(OH)$_2$D$_3$	MCF-7	1, 10 o 100nM, 2 days	25

AUTHOR & YEAR of PUBLICATION	MICRONUTRIENT STUDIED	MODEL	DOSIS & TIME	REFERENCE
NOLAN 1998	1,25$(OH)_2D_3$	MCF-7 variants resistant to ER antagonists	100nM, 96h	23
JAMES 1996	1,25$(OH)_2D_3$	MCF-7 & MNU rats	10^{-8}M, 6 days	21

Table S2. Vitamin D, pre-clinical studies

AUTHOR & YEAR of PUBLICATION	MICRONUTRIENT STUDIED	MODEL	DOSIS & TIME	REFERENCES
ANISIEWICZ 2018	CALCITRIOL and low-calcemic analogs	4T1 Mice	0.5 ug/kg (3/w from day 7 to day 33)	33
CHEN 2018	Vit D	1α(OH)ase$^{-/-}$ Mice	3/w subcutaneous injections with 1.25$(OH)_2D_3$.1μg/kg per mouse	38
LI 2016	Vit D	PyMT-MMTV Mice	1,25$(OH)_2$D : 0,1nM & 100nM or 25(OH)D: 1nM & 100nM	40
SWAMI 2016	Vit D	C57BL/6 mice with cells from Mmtv-wnt1 mice injected	6 weeks	25
WILLIAMS 2016	Vit D	Balb/c mice with cells from Mmtv-wnt1 injected	2 Diets (w/wo Vit D). 10 weeks	36
GARCÍA-QUIROZ 2014	Calcitriol	Nu/Nu mice with T-47D cell xenotransplant and from primary BC cell culture	Calcitriol intraperitoneal injections 0.03ul/g/body weight. 3 weeks.	39
ROSSDEUTSCHER 2014	Vit D	MMTV-PyMT mice	low diet (25 IU/kg) or normal (1000 IU/Kg) Vit D. From week 3 until sacrifice	35
NOLAN 1998	1,25$(OH)_2D_3$	Variants of MCF-7 resistant to ER antagonist & nu/nu mice	100nM 1,25$(OH)_2D_3$ for 96h	23
JAMES	1,25$(OH)_2D_3$	MCF-7 & MNU rats	10^{-8}M. 6 days	21

AUTHOR & YEAR of PUBLICATION	MICRONUTRIENT STUDIED	MODEL	DOSIS & TIME	REFERENCES
1996				

Table S3. Vitamin D, clinical studies

AUTHOR & YEAR of PUBLICATION	MICRONUTRIENT STUDIED	MODEL	DOSIS & TIME	REFERENCES
MADDEN 2018	Vit D	Supplementation after diagnosis (January 2001-December 2011). Ireland cancer data bases were analyzed & associated to Vit D medical prescriptions	10µg/day since death or since December 2011	9
PEPPONE 2012	Vit D	Peripheral blood test from 224 women with BC	1000 IU/day or 50000 IU/w. 8-16 w	37
AMIR 2010	Vit D_3	38 women with BC & bone metastasis	10000UI/day & 1000mg calcium. 4 months	34
SHIN 2018	Vit D	Korean women diagnosed with BC and with resection surgery	Measure of blood [25(OH)D]	41
YAO 2017	Vit D	25OHD measure in serum in 1666 women with BC	Women followed for 12, 24, 48, 72 & 96 months	42
THANASITTHICHAI 2015	Vit D	Serum levels in 200 women with BC	Serum levels by HPLC	43
PALMIERI 2006	Vit D (25(OH)D)	279 Caucasian women with BC	Serum levels	44
MAWER 1996	$1,25(OH)_2D_3$	129 women with BC	Serum levels	45

Table S4. Metals

AUTHOR & YEAR of PUBLICATION	MICRONUTRIENT STUDIED	MODEL	DOSIS & TIME	REFERENCE
CHAN 2016	Cu	75 women with BC	Patients took a Cu chelate to maintain ceruloplasmin levels 8-17mg/dl for two years or until relapse	60
CIHAN 2011	Several metals	52 women with BC Stage III	Trace elements in hair measured	57
OSTRAKHOVITCH 2004	Cu & Zn	MCF-7 & MDA-MB-231	10 & 25μM $CuSO_4$ or 10 & 25μM $ZnSO_4$ for 4 days	56
JABLONSKA 2017	Se & Fe	Tumoral tissue from 42 women with BC	–	70
CHIFMAN 2017	Fe	Predictive mathematical model	—	67
GARCÍA 2016	Fe	MCF-7 & MDA-MB-231	Fe concentration in cell cultures	69
COOMBS 2015	Fe	4T1mice & MDA-MB-468	Fe chelates in different concentrations for 72h	68
CIHAN 2011	Several metals	52 women with BC Stage III	Trace elements in hair	57
JABLONSKA 2017	Se & Fe	Tumoral tissue from 42 women	-	70
WARRINGTON 2013	Se	BALB/c mice & MCF-7	0.16/ 0.51/ 0.85 & 1.15 ppm in milk casein. 10 weeks	79
GUO 2013	Se	BALB/cByJNarl mice	Se measured in serum 22 days after the injection with tumor cells	78
HARRIS 2012	Se	3146 Swedish women with invasive BC	Nutritional questionnaire with mean 10.6 years before BC diagnosis	77
CIHAN 2011	Several metals	52 women with BC Stage III	Trace elements in hair	57

**MATSUI 2017**	Zinc	MCF-7	Cells supplemented with G & measure intracellular Zn^{2+}	89
**COSTELLO 2016**	Zinc	Human tissue	-	85
**CIHAN 2011**	Various	52 women diagnosed with BC Stage III	Trace elements in hair	57
**KAGARA 2007**	Zinc	Samples BC from 177 women with primary breast tumors & several BC cell cultures	Zn chelation	87

Table S5. Folates

AUTHOR & YEAR of PUBLICATION	MICRONUTRIENT STUDIED	MODEL	DOSIS & TIME	REFERENCE
**HANSEN 2017**	Folic Acid	MMTV-PyMT Transgenic Mice	10mg/kg (high) or 2mg/Kg (normal) for 40 days	98
**MANSHADI 2014**	Folic Acid	Sprague-Dawley Rats	2 mg/kg (control), 5 mg/kg, 8 mg/kg or 10mg/kg FA for 12 weeks	102
**LEE 2012**	B_2, B_6 & Folate	980 women	Nutrition questionnaire when diagnosis	100
**LUBECKA-PIETRUSZEWSKA 2013**	Folic Acid	MCF-7 & MDA-MB-231	Cell cultures with 1mg/l FA For treatments add 4mg/l (low) or 8mg/l (high)	101
**HARRIS 2012**	Folate	3116 women with BC	Nutrition questionnaire	103
**NAUSHAD 2012**	Folate	286 women with BC	Blood test analysis	104
**KOTSOPOULOS 2005**	Folates	Sprague-Dawley (tumors induced by MNU)	0, 2 (control) or 8 mg/kg FA in diet during initiation and promotion of tumors	99

Table S6. Vitamin C

AUTHOR & YEAR of PUBLICATION	MICRONUTRIENT STUDIED	MODEL	DOSIS & TIME	REFERENCE
CHA 2016	Vit C	Balb/c mice without endorse synthesis of Vit C (Gulo$^{-/-}$) & mice with production of human Lp(a)+ Inoculated with 4T1 tumor cells	Several treatments for 6 w	112
NAGAMMA 2014	Vit C & α-tocopherol	42 non-smoking women, 42 non-smoking women with BC & 42 smoking women with BC	Serum levels	116
HARRIS 2013	Vit C	3405 women	Nutritional questionnaire	114
MIKIROVA 2012	Vit C	12 women with BC	Intravenous dose: 7.5g-50g	117
KIM 2006	Ascorbic Acid	MCF-7 cells	1,2,5 or 10 mM for 3 days	113
KHANZODE 2004	Ascorbic Acid	96 women with BC without treatment	Serum levels	115

Table S7. Polyphenols

AUTHOR & YEAR of PUBLICATION	MICRONUTRIENT STUDIED	MODEL	DOSIS & TIME	REFERENCE
NASO 2016	Luteolin	MDA-MB-231 cell cultures	Migration test 4h (8.5μM) Adhesion test 24h (8.5μM)	130
BOUALLAGUI 2011	Hydroxytyrosol	MCF-7 cell cultures	2000, 2200, 2400, 2600, 2800 y 3000 μg/ml on different incubation times	129
SCHINDLER 2006	Flavonoids & Vit E	MDA Cell cultures	0.1, 1 or 100 μM/L 24h	131
MURATA 2004	Soy Isoflavones	MCF-7 cell cultures	10^{-10}-10^{-5}M. 6 days	128

Table S8. Fatty Acid

AUTHOR & YEAR of PUBLICATION	MICRONUTRIENT STUDIED	MODEL	DOSIS & TIME	REFERENCE
FEUERECKER 2012	Lipoic Acid	SkBr3 cell culture & naked mice	SkBr3: 2,5/5/7.5 & 10mM Naked mice: 18.5mg/kg/day (28 days)	142
DOZIO 2010	α-lipoic acid	MCF-7 cell culture	0.25-2.5mM for 24,48 & 72h	141
ROSSI 2008	Lipoic Acid	Mice over-expressing HER2	80, 300 or 500μg/day for life	144
MENENDEZ 2006	α-linolenic Acid	BT-474 & SKBr-3 cell cultures	10 or 20 μM	143

Table S9. Vitamin E

AUTHOR & YEAR of PUBLICATION	MICRONUTRIENT STUDIED	MODEL	DOSIS & TIME	REFERENCE
ALGAYADH 2016	Tocotrienol (Vit E)	MDA-MB-231 & MCF-10A cell cultures	0-20μM/day in fresh medium for 4 days. 0-60μM for acute treatment for 24h. 0-6μM for 4 days for western blot	152
NAGAMMA 2014	Vit C & α-tocopherol	42 non-smoking women, 42 non-smoking women with BC & 42 smoking women with BC	Serum levels	154
ELANGOVAN 2008	δ-tocotrienol	MDA-MB-231	1-20μM in 24, 48 & 72h	153
SCHINDLER 2006	Flavonoids & Vit E	MDA Cell culture	0.1, 1 or 100 μM/L 24h	131

Table S10. Iodine

AUTHOR & YEAR of PUBLICATION	MICRONUTRIENT STUDIED	MODEL	DOSIS & TIME	REFERENCE
ALFARO (2003)	Iodine	Sprague-Dawley rats tumor induced by MNU	0.05% I_2 in drinking water	171
MENDIETA (2019)	Iodine	MCF-7 & MDA-MB-231	200 μM I_2	176
STODDARD (2008)	Iodine/Iodide	MCF-7	Lugol (5%I_2 & 10%KI)<br>1 & 5mM iodine/iodide 48h	174
